# Repellent Activity of Some Essential Oils Against *Simulium* Species in India

**DOI:** 10.1673/031.012.0501

**Published:** 2012-01-22

**Authors:** S Hazarika, Sunil Dhiman, Bipul Rabha, RK Bhola, Lokendera Singh

**Affiliations:** ^1^ Defence Research Laboratory (DRL), P.B No.2, Tezpur (Assam), India; ^2^ Department of Zoology, Gauhati University Guwahati, India

**Keywords:** blackfly, biting activity, protection time, repellency

## Abstract

Use of repellents seems to be most reliable method of personal protection against annoyance and infections associated with haematophagous insects. We have investigated the biting activity of *Simulium* and tested the repellency of five essential oils extracted from *Homalomena aromatica* Schott (Alismatales: Araceae), *Pogostemon heyneanus* Bentham (Lamiales: Lamiaceae), *Citrus aurantifolia* Swingle (Sapindales: Rutaceae), *Vitex negundo* L. (Lamiales: Lamiaceae), and *Ageratum conzoides* L. (Asterales: Asteraceae) on the human volunteers against *Simulium* (blackflies) in three locations of Arunachal Pradesh, India. Blackflies preferred biting legs (> 79%) as compared to hand and face with profound biting activity during 1000–1100 h (> 23%) and 1500 – 1600 h (> 28%). The essential oil extracted from *Homalomena aromatica, Vitex negundo* and *Ageratum conizoides* provided > 2 h protection at 5% concentration and > 5 h protection at 10% concentration in all the three testing locations. The repellency of *Homalomena aromatica, Vitex negundo* and *Ageratum conizoides* essential oils after 6 h application was > 50% at 5% concentration and > 90% at 10% concentration. The study provides evidence for the potential of these essential oils in developing new repellents against blackflies.

## Introduction

**Figure 1.**
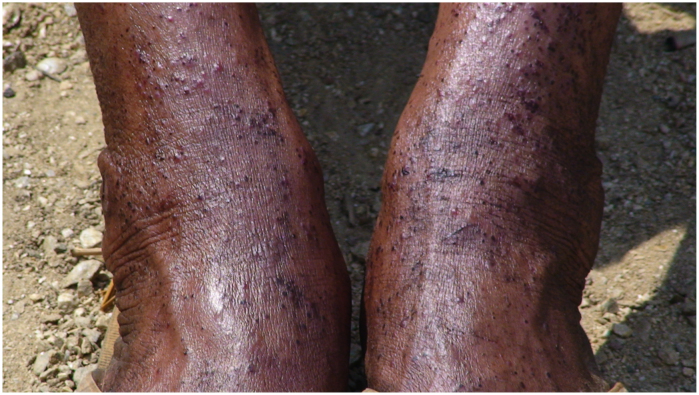
Secondary infection in human legs in Salari of Arunachal Pradesh, India. High quality figures are available online.

Insect transmitted diseases in tropical countries remain a major health threat causing great morbidity every year. *Simulium* species transmit *Onchocerca volvulus* which causes river blindness (onchocerciasis) in African countries (WHO 1995; Aisen et al. 2004). The parasite of onchocerciasis cause itching and disfiguring of skin, nodules on bony areas, serious eye lesions and blindness, therefore producing both public health problems and socio-economic hazards of a considerable magnitude (Opara et al. 2005; Youssefi et al. 2008). In countries like India, where although the parasitic load of *Onchocerca volvulus* is not present, still the vectors bite and crawl on the skin of individual causing intolerable nuisance (Malhotra et al. 1986). Their painful bit leads to the significant amount of blood loss and the wounds can cause secondary infections (Fig. 1) by serving as passage for bacteria, viruses, protozoa and nematodes etc., which the fly may carry on their bodies (Usip et al. 2006).

The eradication of vector borne disease is primarily based on destruction of vector larvae, killing of adult parasite in host's body and preventing vector-host contacts (Killeen et al. 2000; Usip et al. 2006). A number of synthetic chemical agents have been employed to destroy the black flies larvae but epidemiological results don't seem impressive (Walsh 1970; WHO 1995). This effort requires all potential breeding rivers treatment with suitable insecticides. The major problem faced by such control method is that nontarget organisms are also affected leaving a negative feedback on the ecosystem (Malau et al. 2008). Further, large area infested with blackfly larvae makes such control methods more daunting. Control of adult blackflies is most desirable but it is less feasible due to frequent migration and long flight range of vector (Aisen et al. 2004). Thus there exists a need to explore effective repellents in order to make the human host less attractive to black flies (Usip et al. 2006).

Plant derivatives have been demonstrated to possess anti-insects properties (Bhuyan et al. 1974; Tawatsin et al. 2001; Aisen et al. 2004; Usip et al. 2006; Malau and James 2008; Zahir et al. 2009; Kamaraj et al. 2010). However the information on repellent activity of essential oils against blackflies biting is very scanty. The present investigation is aimed at understanding the biting behaviour of adult female blackflies and testing the repellency of some essential oils derived from indigenous plant material for the development of suitable biodegradable repellent against biting black flies.

## Materials and Methods

### Study sites

Repellent trials were carried out in three locations namely Dirang, Salari and Tenga in Arunachal Pradesh of north-east India during 2008–09. Study sites have fast flowing fresh water streams surrounded by coniferous forests. The altitude is > 5,000 ft (mean sea level) with temperature ranging from -2 to 10°C in winters and 10 to 30°C in summers. The inhabitants are agriculture farmers, woodcutters and hunters working from sunrise to sunset making themselves available for blackfly bites throughout the day.

### Test repellents and insects

Essential oils from the rhizome of *Homalomena aromatica* and leaves of *Pogostemon heyneanus, Citrus aurantifolia, Vitex negundo* and *Ageratum conizoides* were extracted by steam distillation method using Clevenger type apparatus in the laboratory. The plants selected in the present study have a history of medical use. Blackflies pupae were collected using the methods of Puri et al. (1932a, 1932b) from different breeding habitats and identified to understand the species composition. *Simulium himalayense* Puri (Diptera: Simulidae), *S. barraudi* Puri, *S. kupari* Datta, *S. indicum* Becher and *S. rufibasis* Brunetti were prevalent during the study period.

### Test procedure

Dilutions of the each essential oil were made to stock in sunflower oil, *Helianthus annus* L. : (Asterales: Asteraceae), v/v at 10% concentration, from which 5%, 7.5% concentrations were prepared. Each trial was conducted using six volunteers (three males and three females) for each concentration of each essential oil in each of the three testing sites. Volunteers aged between 18 to 50 years participated in the study were non-smokers, non-alcoholic and had no known history of allergic reactions to insect bites or herbal oils. The written consents were obtained from each test volunteer prior to the commencing of trial. 1.5 ml dilution of each of three concentrations of each essential oil was applied at a rate of 1 ml per 500 cm^2^ topically on each arm from elbow to finger tips (area approx 750 cm^2^) and 2.5 ml on each leg from knee to foot (area approx 1250 cm^2^) of test volunteers after disinfecting the treatment area skin with alcohol (Das et al. 1985; Usip et al. 2006). Two individuals (one male and female) applied only *Helianthus annus* oil and served as control for each test. Both the control and test volunteers sat still exposing the treated area at about four meters apart from each other during the experiments. The elasped time to the first bite of first two consecutive bites occurring within 30 minutes was recorded as the complete protection time (Das et al. 1985; Usip et al. 2006). The biting frequency (number of flies landing/per subject/minute) was determined every time before starting the trial.

**Figure 2.**
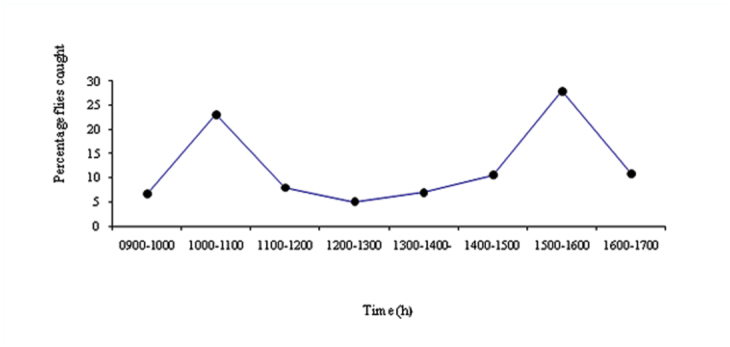
Bimodal biting activity of *Simulium* sp. attracted to human (three sites combined). High quality figures are available online.

**Figure 3.**
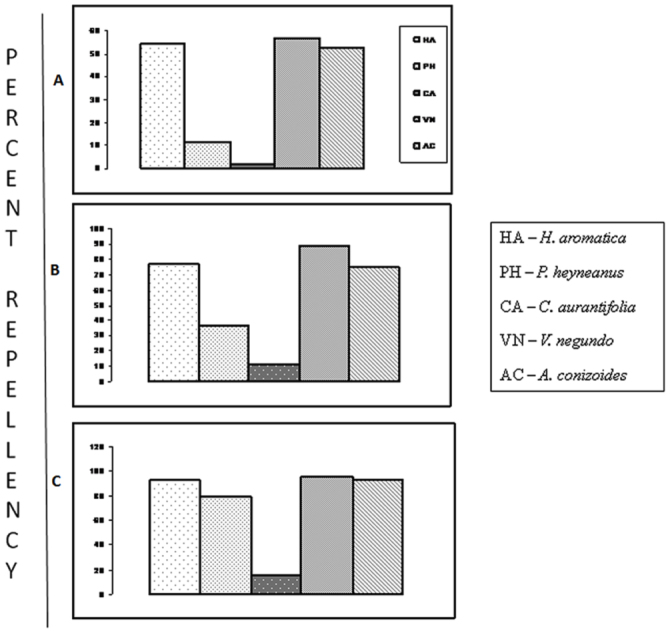
Percent repellency of essential oils after 6 h of application, (A) at 5% conc., (B) at 7.5% conc., and (C) at 10% conc. High quality figures are available online.

During the test period two volunteers sat exposing hands, feet and face without any application to find out the peak-biting hour and body part preference of biting flies. The flies landing on exposed body parts to suck blood were caught before it actually fed by inverting small eppendorff tubes over them and the tubes were labeled to indicate time and date of capture. The process was repeated everyday and every location using different volunteers. The collected flies were stored for future molecular taxonomy studies in the laboratory. Trials were conducted from 0900 hrs to 1700 hrs and the entire study procedure was cleared by the ethical committee of the Defence Research Laboratory, Tezpur India. The study was funded by Defence R&D Organisation, Ministry of Defence, Govt. of India.

**Table 1.**
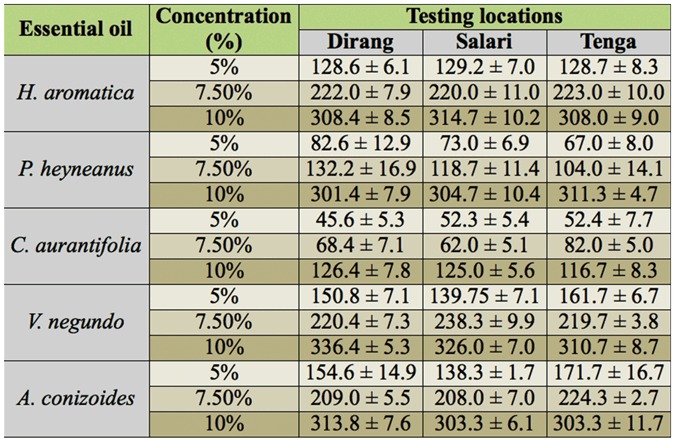
Repellency (average protection time in minutes ± SEM) of five essential oils against *Simulium* sp. at three testing locations in Arunachal Pradesh, India.

### Data analysis

All the data obtained were tested for normality using Kolmogorov and Smirnov method (Stephens 1979). The results obtained were analyzed using student's t-test and analysis of variance (ANOVA) followed by TukeyKramer multiple comparison tests at 95% confidence interval using GraphPad InStat software. Percent repellency was calculated following the methods of Sharma and Ansari (1994) and Yap et al. (1998).

## Results

The biting activity of blackflies is presented in fig. 2. The results indicate that biting flies have significantly higher activity during 15:00 to 16:00 h (115.0 landings/hr ± 5.1, 28.1% landing) compared to 10:00 to 11:00 h (95.0 ± 1.4, 23.2%) (*p* < 0.01, *t* = 3.79) and other biting hours (*p* < 0.01, *F* = 188.7). The flies preferred biting legs (79.1%) rather than hand (17.7%) and face (3.2%) (*P* < 0.0001, *F* = 421.0). The biting pressure at Dirang (3.12 ± 0.2) was significantly high (*P* = 0.0018, *F* = 9.3) than in Salari (2.48 ± 0.3) and Tenga (1.84 ± 0.1).

The repellent activity of five essential oils tested against the biting of black flies in three different locations has been presented in table 1. The results obtained indicated that average protection time (APT) achieved with 10% concentration of each essential oil was significantly higher than the other two concentrations (*P* ≤ 0.002, *F* ≥ 20.29) tested in all the three testing locations. There was no significant difference in the protection time produced by similar concentrations of an essential oil among the three testing locations (*P* ≥ 0.063, *F* ≤ 3. 97). However the protection time produced by similar concentrations of all the tested essential oils in all the testing locations differs significantly (data pooled). In 5% concentration, *A. conizoides* essential oil protection time, 154.8 ± 8.4 minutes (APT ± SEM) was higher (*P* < 0.0001, *F* = 69.59) but did not differ significantly from *V. negundo* (*P* > 0.05), whereas in 7.5 % concentration, protection time was higher in *V. negundo* (225.6 ± 4.8) (*P* < 0.0001, *F* = 152.22) but was similar (*P* = 0.15, *F* = 2.04) to that in *A. conizoides* and *H. aromatica*. In 10% concentration, *V. negundo* essential oil protection time was 326.55 ± 4.8, which was significantly higher (*P* < 0.0001, *F* = 333.24) but did not differ significantly from *H. aromatica* (*P* > 0.05). No bite was observed upto 2 h with 5% and upto 3 h with 7.5% topical application of *H. aromatica, V. negundo* and *A. conizoides* essential oils. However in *P. heyneanus* and *C. aurantifolia* the repellency fell to 88.42% and 85.26% respectively at 5% concentration after 2 h. After 6 h of application, *H. aromatica, V. negundo* and *A. conizoides* essential oils at 5% concentration produced > 50% repellency, whereas at 10% concentration > 90% repellency was achieved [Fig. 3 a, b, c]. The control treatment could provide protection up to 30.8 ± 2.5 minutes. There was no skin irritation, hot sensations or rashes observed on the legs or arms of test volunteers treated with essential oils during six months after the study.

## Discussion

The plant based repellents may have good role in reducing the biting nuisance and infections by preventing the man-fly contact (Curtis 1992; Fradin 1998; Fradin and Day 2002; Usip et al. 2006; Spero et al. 2008) and to preclude any adverse effect that could emanate from the use of synthetic repellents (Osimitz et al. 1995; Goodyer and Behrens 1998). Many plants oils and material have been tested as potential insect repellents (King 1954; Quarles 1996; Tawatsin et al. 2001) and are available in the market either in single or in combination with other. The results of present study showed that topical application of essential oils extracted from the plants significantly reduced the biting rate of Simuliids species. The extracts of *Aristolochia indica, Cassia angustifolia, Diospyros melanoxylon, Dolichos biflorus, Gymnema sylvestre, Justicia procumbens, Mimosa pudica* and *Zingiber zerumbet* have been used as mosquito repellent and produced good results (Kamaraj et al. 2010), also the volatile oils of some plants have been proved effective as repellent against blackflies (Usip et al. 2006).

In the present investigation essential oil of *Homalomena aromatica, Vitex negundo, Ageratum conizoides* at 10% concentration repelled the blackflies for >5 h during their peak biting hours, which indicates that topical application of these essential oil could provide effective repellency for 5 to 6 h during the active biting period of blackflies. In other studies the N, N diethyl-meta-toluamide (DEET) and p-menthane-3, 8-diol—based repellent have produced 8–10 h protection time (Fradin and Day 2002; Trongtokit et al. 2002) against mosquito species whereas 2–3 h protection was achieved by a plant—based repellent (Zahir et al. 2009; Kamaraj et al. 2010). However in our study we could achieve > 2 h and > 5 h repellency at 5% and 10% concentration respectively with some of the tested essential oils, which suggests that essential oils extracted from *Homalomena aromatica, Vitex negundo* and *Ageratum conizoides* are very effective against blackflies in northeast India. Our findings are similar to those achieved elsewhere (Bhuyan et al. 1974; Kumar et al. 1984; Das et al. 1985; Malhotra et al. 1986; Debboun et al. 2000; Usip et al. 2006). Prevention of vector borne diseases lies on the use of protective wearing in parasite infested areas and applying efficient repellent on exposed body parts (Usip et al. 2006). Our findings agreed with Aisen et al. (2004) and Usip et al. (2006), who showed that volatile oils have produced protection for more than 3 h. However the studies carried over by Das et al. (1985) revealed that 20% dimethyl phthalate and DEET are capable of producing protection for 6 and 7 h respectively whereas both at 10% concentration could produce protection time > 4 h. The efficiency of a repellent depends on various factors such as age, sex, vector species, temperature, rainfall and wind velocity etc. (Fraddin 1998; Golenda et al. 1999). These factors singly or in combination may result in varied repellency of an extract or oil even in a same individual. Therefore a given repellent may not protect all the users equally and variation may occur in the ability of repellent to protect different subjects (Fradin and Day 2002). The repellent times achieved by an essential oil should be taken as an indication of its effectiveness and not as absolute value.

Biting activity of blackflies occurs in bimodal fashion being high in morning, as it was recorded high in morning hours and early evening hours. In the previous studies, Kutin et al. (2004), Opara et al. (2005) and Usip et al. (2006) have found the similar results. There was no biting activity recorded during 1230–1330 h by Usip et al. (2006) whereas in the present study 5–7 % biting activity was recorded during this period. Peak biting time of black flies may correspond to the high outdoor human activities in the morning and early evening hours, making human available to the flies (Usip et al. 2006). The biting activity may be related to the sunshine, which could be an important factor in the blackfly activity, or to the availability of hosts. Blackflies preferred biting the exposed legs in comparison to the exposed face and hands of human. This may be primarily due to the lesser mobility and activity in lower human body parts. The face is most active exposed part of the human body followed by hands, which may have reduced the chances of bites on these parts. Further legs are near the ground level which may also enhance the chance of bites.

Various reports on the use of plant based materials for the management and control of various pest and vectors of disease agents have been documented. Herbal repellents provide a safe, eco friendly and inexpensive means to protect individuals from vector biting (WHO 1995). The bio-chemical substances in plant based repellent are not accumulated in the food chain as do in other synthetic chemical compounds which are the major cause of environmental pollution (Malau and James, 2008). Further the testing of new compounds as insect vector repellent is of immense importance in the preview of resistance development.

## Conclusion

In the present study we have found that the blackflies are more active during morning hours and early evening hours and prefer biting legs rather than hands or face. The potential plants based essential oils tested in the present study provided protection time > 5 h at 10% concentration, hence are suitable candidates as blackflies repellents. The essential oils appeared to have no toxic effects in this study and a history of herbal medicine use indicates safety, which is further confirmed here by the dermatological test carried on the mice model. The present results have clearly demonstrated the possibility of transforming the potential natural products into commercial repellent after testing in different geo-climates and blackflies species.
